# Soil Properties and Microbial Community Assemblages in Response to Plastic Film Mulches with Divergent Degradation Characteristics

**DOI:** 10.3390/microorganisms14030553

**Published:** 2026-02-28

**Authors:** Guiliang Wang, Yulin Li, Xu Pan, Aofei Li, Juanjuan Wang, Li Yin, Xiaoping Zeng, Xiaoqing Qian

**Affiliations:** 1 Key Laboratory of Arable Land Quality Monitoring and Evaluation, Ministry of Agriculture and Rural Affairs, Yangzhou University, Yangzhou 225127, China; wgl0520@126.com (G.W.); mz120231319@stu.yzu.edu.cn (Y.L.); mz120241376@stu.yzu.edu.cn (X.P.); 18749206734@163.com (A.L.); qianxq@yzu.edu.cn (X.Q.); 2 Yangzhou Jiangdu District Comprehensive Agricultural Technology Service Center, Yangzhou 225200, China; 3 Jiangsu Provincial Agricultural Technology Extension Station, Nanjing 210036, China; mjj110929@163.com

**Keywords:** plastic film mulching, broccoli, soil fertility, microbial community, degradation

## Abstract

To identify a suitable plastic film type for broccoli cultivation in the subtropical humid region of southern China, a field experiment was conducted with four treatments, including no film control (CK), reinforced polyethylene film (RF), biodegradable film PBAT + starch (BDF1), and biodegradable film PBAT + PLA (BDF2). Soil physiochemical properties, temperature and humidity dynamics, microbial community structure, and film degradation status were investigated. The results showed that the RF treatment improved available P and K contents, while the BDF2 treatment significantly increased soil organic matter, NH4^+^-N, water-soluble Ca^2+,^ and Mg^2+^ contents. The soil temperature followed the order of RF > BDF1 > BDF2 > CK, and the humidity was BDF1 > RF > CK > BDF2, with RF treatment showing a more stable soil temperature, while BDF2 treatment fluctuated the most. There were no significant differences in bacterial diversity among the treatments, while the highest fungal diversity was observed in the BDF2. Water-soluble Mg was the key factor driving the changes in microbial community structure (*p* < 0.05). The film degradation rate followed BDF2 > BDF1 > RF. Collectively, RF is suitable for targeting short-term yield improvement, while BDF2 has significant advantages in sustainable cultivation in the long-term.

## 1. Introduction

Broccoli (*Brassica oleracea var. italica*), as an important vegetable in the Brassicaceae family with both nutritional and economic value, has become one of the mainstream vegetable varieties in the global consumer market due to its rich vitamin C, dietary fiber, and various antioxidant compounds [1]. As a major producer and consumer of broccoli in the world, China’s planting area reached 0.104 million hectares in 2024 [2]. However, under open-field conditions, soil hydrothermal conditions are easily affected by the seasonal uneven precipitation and large temperature fluctuations. The traditional open-field cultivation cannot sustain the yield and quality of broccoli, restricting the sustainable development of the broccoli industry [3].

Plastic film mulching improves the soil microenvironment through a physical barrier, and has been widely used in vegetable production with significant yield increases [4,5]. However, the long-term effects of films with different materials on the soil ecosystem vary significantly. Traditional reinforced polyethylene film (RF) with excellent heat preservation and humidity retention properties can increase crop yield, but it is difficult to degrade. Long-term use may lead to the accumulation of microplastics in the soil, damage the soil structure, and threaten ecosystems [6,7]; biodegradable films made from poly (butylene adipate-co-terephthalate) (PBAT), polylactic acid (PLA), starch, etc., can be degraded into CO_2_ and water through microbial activities, and have become an important alternative for addressing “white pollution” in farmland [8,9,10].

The potential consequences of biodegradable plastics in soil ecosystems are complex and regulated by multiple factors, such as crop, soil type, climate conditions, and film materials [11]. Li et al. [12] found that biodegradable film reduced soil pH and organic matter content in cabbage fields, but had no significant effect on soil available nutrients; Shan et al. [13] pointed out that plastic film mulching significantly increased nitrate nitrogen in broccoli and pepper fields, but decreased it in garlic fields. Mazzon et al. [14] concluded that soil physicochemical characteristics influenced the impact of biodegradable plastic films. Soil microorganisms play a key role in soil nutrient cycling and interact with plastic films through various pathways, but the impacts of mulch films on soil microbial community structure and function vary greatly due to multiple factors [15]. Li et al. found that the PBAT/PLA plastisphere had lower bacterial α-diversity, with enriched taxa that potentially degrade plastic polymers such as *Proteobacteria* and Actinobacteria [16]. Similarly, Liu et al. [17] also observed enriched degradation-related microorganisms (e.g., *Bacillales*, *Burkholderiales*, and *Actinomycetales*) under biodegradable film mulching, which also decreased the evenness of soil fungi and archaea and the abundance of potential pathogenic microorganisms. Wang et al. [18] noted that the PBAT application significantly increased the abundance of certain microorganisms like Aspergillus and Penicillium.

Despite the growing focus on biodegradable films as alternatives to conventional polyethylene (PE) films, knowledge gaps remain regarding their impacts on soil health [19]. Current studies mostly focus only on chemical indicators or biological traits [20,21], while research on the synergistic responses of microbial communities and the relationship between film degradation and soil fertility evolution remains limited [22], which is a critical consideration for selecting appropriate plastic mulch films. Therefore, this study aims to link film degradation characteristics with soil hydrothermal dynamics, nutrient changes, and microbial community assemblages, providing a comprehensive evaluation of film performance and ecological effects.

Yangzhou City of Jiangsu Province is a major broccoli-producing area in southern China. Long-term use of traditional PE films in this region has shown signs of microplastic accumulation, while the application of biodegradable films lacks systematic data support. This study was conducted to clarify the effects of different plastic films on soil fertility and microbial community composition in the subtropical humid region of southern China, thereby providing a reference for selecting films for green broccoli cultivation. The objectives were: (1) to compare the effects of different types of plastic mulch films on soil hydrothermal dynamics and physicochemical parameters; (2) to investigate the response of soil bacterial and fungal communities to different mulch films and the key soil parameters driving microbial community changes; and (3) to evaluate the degradation characteristics of different films and their comprehensive suitability for broccoli cultivation.

## 2. Materials and Methods

### 2.1. Experimental Site

The experiment was conducted in the Modern Agricultural Industrial Park of Xiaoji Town, Jiangdu District, Yangzhou City, Jiangsu Province (32°62′ N, 119°74′ E), which belongs to a subtropical humid monsoon climate. It has an annual precipitation of 1000–1200 mm, an annual evaporation of 1200–1300 mm, and an annual average temperature of 14–15 °C. The experimental period was from September 2023 to January 2024. The soil was classified as a luvo-aquic soil according to the Chinese Soil Taxonomy, with a gray color. It developed from parent material of the Yangtze River, forming on fluvial floodplain deposits, with a cultivation history of over 30 years (mainly under rotation of vegetables and wheat). The soil profile has a distinct A horizon (0–20 cm) and C horizon (below 60 cm). The background values of soil properties were as follows: pH 7.98, total organic matter 15.35 g/kg, nitrate nitrogen 24.82 mg/kg, ammonium nitrogen 10.75 mg/kg, available phosphorus 30.6 mg/kg, available potassium 59.6 mg/kg, water-soluble calcium 101.79 mg/kg, water-soluble magnesium 27.37 mg/kg, available iron 17.39 mg/kg, available manganese 2.8 mg/kg, available copper 1.99 mg/kg, available zinc 5.22 mg/kg, available boron 0.86 mg/kg, and available molybdenum 0.05 mg/kg.

### 2.2. Experimental Design

A completely randomized design (CRD) was adopted in the experiment, with four treatments: no film control (CK), reinforced polyethylene film (RF), biodegradable film No.1 (BDF1), and biodegradable film No.2 (BDF2). The plot size was 4 m (width) × 40 m (length), and 1 m isolation rows were set between plots to avoid mutual interference. Each treatment had three replicates, resulting in a total of 12 plots. The characteristics of the tested films are shown in Table 1. Note that the RF (0.018 mm) was thicker than the two biodegradable films (0.01 mm), which may affect thermal conductivity, durability, and degradation behavior—this limitation is discussed in detail in Section 4. The broccoli variety was “Youxiu”, a variety suitable for local conditions. The seedlings were transplanted at the 5-leaf stage, with a planting density of 30 cm × 40 cm.

Bio-organic fertilizer (Maofeng) was applied at a rate of 4500 kg/ha as base fertilizer. Field management (irrigation, pest control, etc.) followed conventional farming practices. Flood irrigation was carried out to maintain soil humidity at 60–80% of field capacity.

### 2.3. Sampling and Determination

#### 2.3.1. Temperature and Humidity Monitoring

After broccoli transplanting, soil temperature and humidity were recorded using Jingchuang GSP-6 recorders. A monitoring point was set at the soil surface (0 cm) and at 10 cm depth under the film in each plot, totaling 24 monitoring points (4 treatments × 3 replicates × 2 depths). Data were recorded once per hour until harvest, and average daily values were calculated for subsequent analysis.

#### 2.3.2. Soil Properties

At the harvest stage (January 2024), 0–20 cm soil samples were collected. Soil cores (3 cm in diameter) from five sampling points were collected from each plot and mixed into one composite sample. The samples were divided into two portions: one was stored frozen at −80 °C for microbial diversity determination; the other portion was air-dried, ground, and sieved. For the determination of soil organic matter (SOM), the soil was sieved through a 0.15 mm sieve, and for other parameters, a 0.85 mm sieve was used.

Soil pH was determined by the potentiometric method (soil-water ratio 1:2.5); SOM was determined by the potassium dichromate volumetric—external heating method; NO_3_^−^-N was determined by the ultraviolet spectrophotometric method; NH_4_^+^-N was determined by the indophenol blue colorimetric method; available phosphorus (AP) was determined by the molybdenum antimony anti-colorimetric method; available potassium (AK) was determined by the flame photometric method; water-soluble calcium (WSCa) and magnesium (WSMg) were determined by the atomic absorption spectrophotometric method; available iron (AFe), manganese (AMn), copper (Acu), and zinc (Azn) were determined by the DTPA extraction—atomic absorption spectrophotometric method; available boron (AB) was determined by the curcumin colorimetric method; available molybdenum (AMo) was determined by the potassium thiocyanate colorimetric method [23].

For soil microbial diversity determination, the soil DNA was extracted using the MoBio PowerSoil DNA Isolation Kit (QIAGEN Inc., Valencia, CA, USA). PCR amplification was performed on the V3–V4 fragment of the bacterial 16S rRNA gene (primers 338F/806R) and the fungal ITS sequence (primers ITS1F/ITS1R). After purification and quantification, the PCR products were sequenced on the Illumina MiSeq platform (Shanghai Maji Biomedical Technology Co., Ltd., Shanghai, China), and the detailed procedures were referred to Wang et al. [24].

#### 2.3.3. Assessment of Plastic Film Degradation

The film was photographed and sampled regularly. The degradation level was first determined visually [25], with the classification standard revised as follows: level 0 (no degradation, intact film), level 1 (initial degradation, small cracks of 1–2 cm appear), level 2 (moderate degradation, more than 25% of the film shows cracks and fragmentation), level 3 (severe degradation, large cracks of 10–25 cm), level 4 (advanced degradation, film broken into small pieces), and level 5 (complete degradation, no visible film remaining). The broken film pieces in the field were collected, rinsed repeatedly with sterile water, dried at 30 °C, and observed under a scanning electron microscope (SEM) to further reveal the degradation characteristics of the plastic films.

### 2.4. Data Analysis

Microsoft Excel 2016 and Origin 2021 were used for data analyses and plotting; IBM SPSS Statistics 19 was used for statistical analysis. One-way analysis of variance (ANOVA) with the LSD multiple comparison test was used to test the differences between treatments (*p* < 0.05). Microbial data analysis was performed on the Majorbio scientific research service platform (https://www.majorbio.com/web/ucenter/personal/account-info/index (accessed on 13 October 2025)). The species classification databases were SILVA 138(16S) for bacteria and UNITE 8.0(ITS) for fungi. The USEARCH11-uparse algorithm was used for clustering. The high-quality sequences were clustered into operational taxonomic units (OTUs) at a 97% similarity threshold; alpha diversity indices (Ace, Chao, Shannon, Simpson) were calculated based on OTU data; beta diversity analysis was used to compare community similarity between treatments. Alpha diversity, beta diversity, community composition, non-metric multidimensional scaling (NMDS), redundancy analysis (RDA), and correlation heatmaps were performed on the platform. RDA was used to identify the relationship between soil physicochemical properties and microbial community structure, with significance tested by permutation test (999 permutations).

## 3. Results

### 3.1. Soil Fertility Characteristics

As shown in Table 2, the soil pH value of each treatment was between 8.04 and 8.19, with no significant difference between treatments. The BDF2 treatment significantly increased the soil organic matter content, which was 13.8% higher than that of CK. Both RF and BDF2 treatments significantly increased the contents of available phosphorus and available potassium. In contrast, the BDF1 treatment had no significant effect on the contents of major nutrients, N, P, and K. The BDF1 treatment had the highest available Fe and available Mn, although the difference was not significant. The contents of water-soluble Ca and Mg in all film mulching treatments were significantly higher than those in CK, with increase rates of 34.0% and 68.2%, respectively, in the BDF2 treatment.

### 3.2. Soil Temperature and Humidity

**Figure 1:**
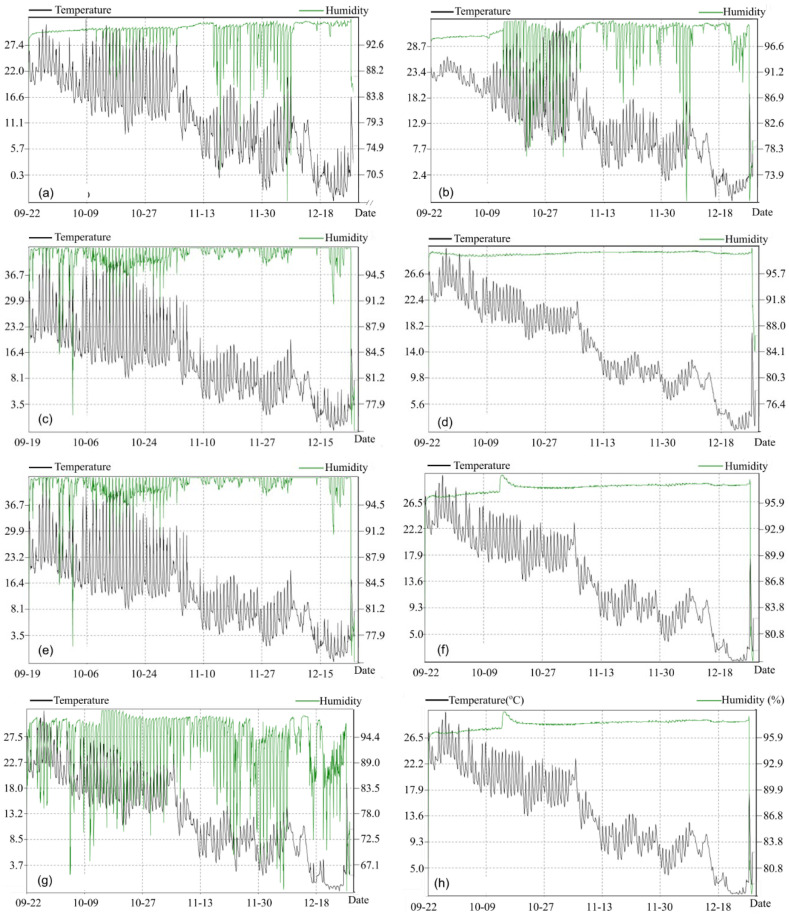
Changes in soil temperature and humidity at the surface and 10 cm underground under different treatments: (**a**) CK surface; (**b**) CK underground; (**c**) RF surface; (**d**) RF underground; (**e**) BDF1 surface; (**f**) BDF1 underground; (**g**) BDF2 surface; (**h**) underground; the left abscissa is the date, the left ordinate is temperature (°C), and the right ordinate is humidity (%).

The patterns of soil temperature and humidity at the surface and 10 cm under the film of different treatments are shown in Figure 1. The soil temperature generally decreased with time during the experiment. The temperature fluctuation of CK was the largest (with a surface temperature variation coefficient of 18.7%), and that of RF was the smallest (variation coefficient of 12.9%). The heat preservation effect was in the order of RF > BDF1 > BDF2 > CK. The temperature change at 10 cm under the film was consistent with that at the surface, with a relatively smaller fluctuation range. The average temperatures of the RF and BDF2 treatments were 2.3 °C and 0.8 °C higher than those of CK.

In terms of soil humidity, CK had direct exchange with atmospheric water vapor, resulting in the largest humidity fluctuation (variation coefficient of 12.3%); the soil humidity under film mulching was more stable than that in CK, and the humidity retention effect was BDF1 > RF > CK > BDF2. The average surface humidity of the BDF1 treatment was 89.2%, which was 6.5% higher than that of CK, while the average surface humidity of the BDF2 treatment was lower than that of CK, with the largest diurnal humidity difference.

### 3.3. Soil Microbial Community Structure

#### 3.3.1. Microbial Diversity

As shown in Table 3, there were no significant differences in bacterial alpha diversity indices (Ace, Chao, Shannon, Simpson) between treatments. The CK treatment had the highest bacterial richness (Ace index), and the BDF1 treatment had the highest evenness (Shannon index). The fungal alpha diversity of the BDF2 treatment was significantly higher than that of other treatments, and its Ace index and Chao index were significantly higher than those of RF and CK (*p* < 0.05). There were no significant differences in the evenness index of fungi between treatments.

#### 3.3.2. Composition of Soil Bacterial and Fungal Communities

**Figure 2:**
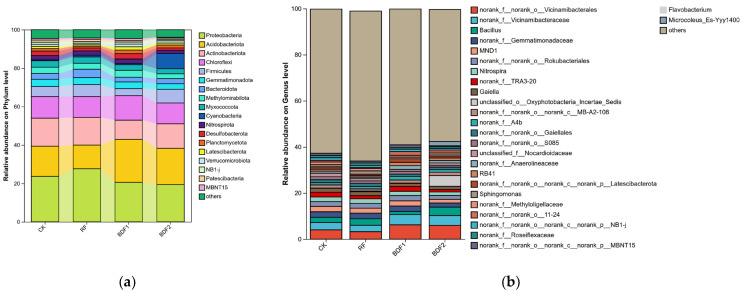

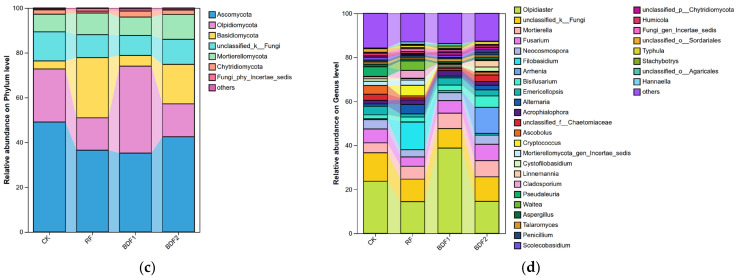
Soil microbial community composition under each treatment (relative abundance > 1%). (**a**) bacterial phylum level; (**b**) bacterial genus level; (**c**) fungal phylum level; (**d**) fungal genus level.

The high-throughput sequencing of the 16S rRNA gene revealed 19 bacterial phyla with a relative abundance > 1% in each treatment (Figure 2). The dominant phyla were *Proteobacteria* (25.3–32.9%), *Acidobacteriota* (18.8–25.4%), Chloroflexi (10.2–14.7%), *Actinobacteriota* (8.5–11.3%), and Firmicutes (6.8–9.2%). Compared with CK, the abundance of *Proteobacteria* in RF and BDF1 treatments increased by 7.66% and 4.12%, respectively, while that in BDF2 treatment decreased by 0.03%; the abundance of *Acidobacteriota* in all film mulching treatments showed a decreasing trend.

At the bacterial genus level (Figure 2b), the dominant genera included *norank_f_norank_o_Vicinamibacterales*, *Flavobacterium*, *norank_f_Vicinamibacteraceae*, etc. The abundance of *Arrhenia* in the BDF2 treatment was relatively high (11.85%), and the abundance of *Filobasidium* in CK was 12.55%. There were no significant differences in other dominant genera between treatments.

*Ascomycota* was the most dominant fungal phylum (35.2–62.88%), followed by *Olpidiomycota* (4.65–38.82%) (Figure 2c). The abundance of Basidiomycota in the BDF1 treatment was significantly higher than that in other treatments (*p* < 0.05). At the genus level, *Olpidiaster* had a high relative abundance in the soil of each treatment, with the order of BDF1, RF, BDF2, and CK; other dominant genera included *Humicola*, *Mortierella*, *Fusarium*, etc., with no significant differences between treatments.

**Figure 3:**
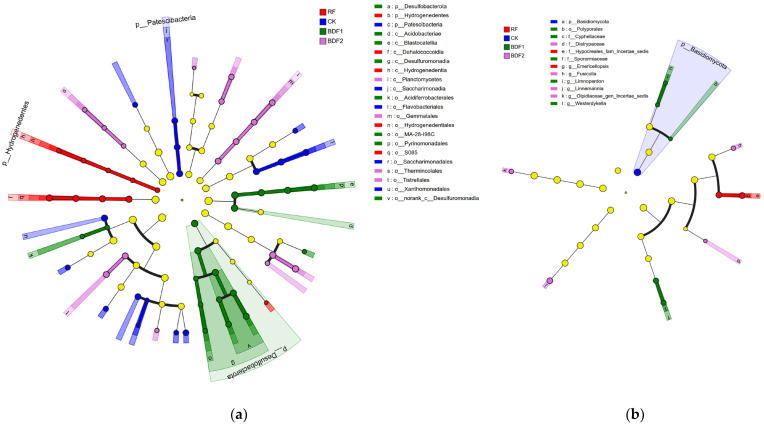
LEfSe analyses of soil microbial communities under each treatment. (**a**) Bacteria; (**b**) fungi; nodes of the same color indicate microbial groups significantly enriched in the corresponding group, light yellow nodes indicate groups with no significant difference, and the diameter of the circle is proportional to the abundance of the taxon.

LEfSe multi-level species difference discrimination was used to analyze the biomarker species in the soil of each treatment (Figure 3). At the bacterial phylum level, *Desulfobacterota*, *Hydrogenedentes*, and *Patescibacteria* contributed greatly to the differences between treatments, which were mainly enriched in BDF1, RF, and CK. With regard to fungi, only Basidiomycota in the BDF1 treatment contributed significantly to the differences between treatments (*p* < 0.05).

#### 3.3.3. Relationship Between Soil Microbial Community and Soil Properties

**Figure 4:**
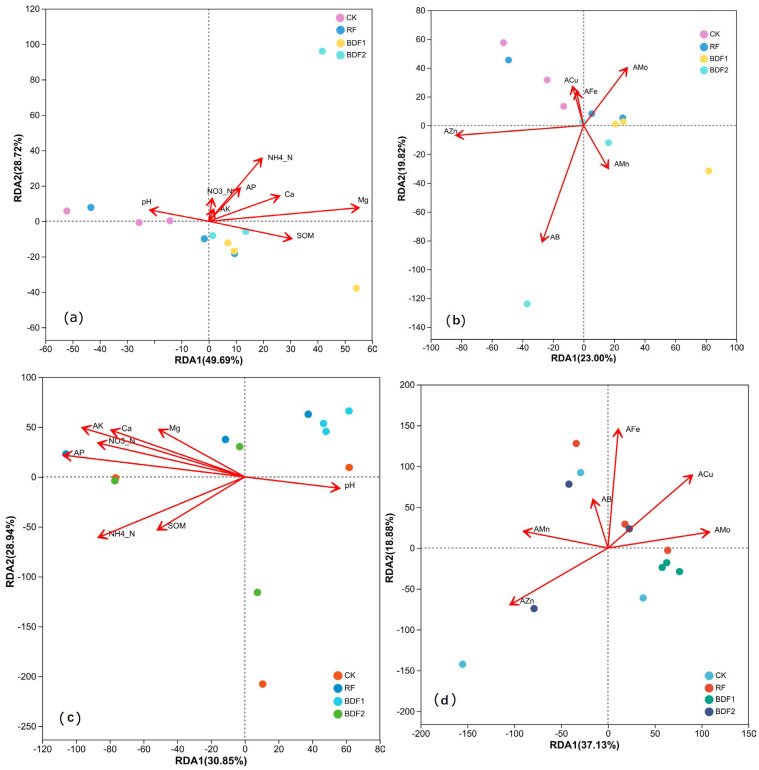
RDA of different treatments. (**a**,**b**): RDA of soil properties and bacterial community composition; (**c**,**d**): RDA of soil properties and fungal community composition. SOM: soil organic matter; NO_3_^−^-N: nitrate nitrogen; NH_4_^+^-N: ammonium nitrogen; AP: available phosphorus; AK: available potassium; Ca: water-soluble calcium; Mg: water-soluble magnesium; AFe, AMn, ACu, AZn, AB, and AMo refer to available iron, manganese, copper, zinc, boron, and molybdenum, respectively.

The results of RDA are shown in Figure 4. Among all soil parameters, water-soluble magnesium had the greatest impact on the microbial community structure (*p* < 0.05) and was negatively correlated with overall microbial community variation. The effects of pH, SOM, and available Cu were also prominent, but not significant. pH, available Fe, available Cu, and available Mo were positively correlated with the microbial community structure of CK and negatively correlated with the changes in the microbial community structure of BDF2. Available B, NH_4_^+^-N, water-soluble Ca, available P, etc., were all negatively correlated with the overall variation in the microbial community structure.

Correlation analysis results showed (Table 4 and Table 5) that water-soluble magnesium was significantly negatively correlated with seven dominant bacterial phyla, including *Proteobacteria*, *Nitrospirota*, *Gemmatimonadota*, etc., and significantly positively correlated with five bacterial phyla, including *Acidobacteriota*, *Planctomycetota*, *Verrucomicrobiota*, etc. *Acidobacteriota* was positively correlated with organic matter, nitrate nitrogen, ammonium nitrogen, available phosphorus, and available potassium, while *Proteobacteria* and *Actinobacteriota* were negatively correlated with these indicators. With regard to fungi, *Ascomycota* was significantly positively correlated with AK, Basidiomycota was significantly positively correlated with available Zn, and significantly negatively correlated with available Mo.

### 3.4. Plastic Film Degradation

**Figure 5:**
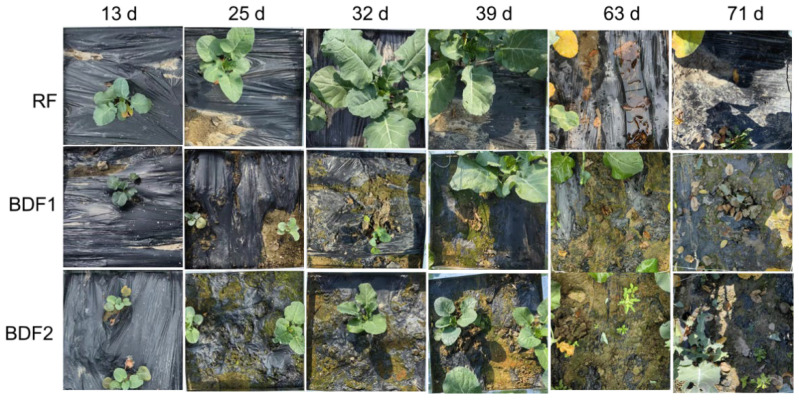
Film degradation at different times.

The results of film degradation classification showed (Figure 5) that no obvious degradation was observed in the films of all treatments at 13 d (level 0); at 25 d, BDF1 reached level 1 and BDF2 reached level 2; at 32 d, BDF1 reached level 2 and BDF2 reached level 3; at 39 d, RF reached level 1, BDF1 remained at level 2, and BDF2 maintained level 3; at 63 d, BDF1 reached level 3, and BDF2 reached level 4; at 71 d, RF remained at level 1, BDF1 remained at level 3, and BDF2 remained at level 4.

**Figure 6:**
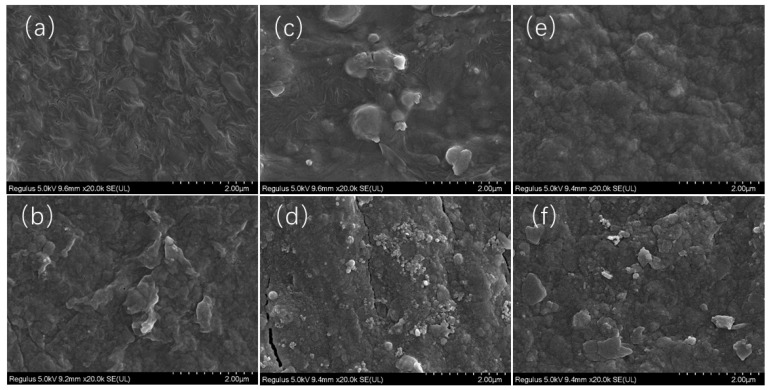
Scanning electron microscope images of film surfaces (2.00 μm). (**a**) RF before; (**b**) RF after; (**c**) BDF1 before; (**d**) BDF1 after; (**e**) BDF2 before; (**f**) BDF2 after.

SEM analysis showed (Figure 6) that the surface of the original film was smooth and intact; after the experiment, the surface of RF became rough, with wrinkles and grooves, but no obvious rupture; the surface of BDF1 had obvious cracks and enriched small particles; and the surface of BDF2 was severely broken, forming a large number of fragments. Overall, the film degradation rate was BDF2 > BDF1 > RF.

## 4. Discussions

Soil temperature and humidity stability are critical for crop growth, and film characteristics (composition, thickness, degradability) directly determine their regulatory capacity [11,26,27]. In this experiment, RF, composed of polyethylene with a greater thickness (0.018 mm), maintained the most stable soil temperature (variation coefficient 12.9%) due to its durable physical barrier, which effectively intercepts solar radiation and reduces heat loss. Contrary to previous studies showing that biodegradable mulch can achieve soil temperature and humidity conditions comparable to conventional PE film [28], this study indicates differences in hydrothermal regulation. A key confounding factor here is the thickness difference between RF (0.018 mm) and the biodegradable films (0.01 mm). Thicker films generally have lower thermal conductivity and higher durability, which may contribute to RF’s superior heat preservation independently of polymer composition. This limitation highlights the need for future studies using films of comparable thickness to isolate the effects of material composition. In contrast, BDF2 (PBAT + PLA) exhibited the largest temperature and humidity fluctuations, which were closely linked to its rapid degradation. As the film degrades, its physical barrier function weakens, increasing soil exposure to atmospheric temperature and humidity variations. Di Mola et al. [29] also proposed that the soil temperature under degradable film was lower than that under ordinary film, suggesting that the heat preservation stability of degradable film needs to be further optimized in combination with the needs of the crop growth period.

Film mulching regulates soil nutrient availability through direct (e.g., nutrient release during degradation) and indirect (e.g., modifying microbial activity) pathways. RF had the most significant effect on improving AP and AK, which was closely related to its excellent water retention performance [30]. In contrast, Liu et al. [31] found that degradable films can effectively increase soil AP, potentially by promoting the activity of phosphorus-mineralizing microorganisms. However, biogenic microplastics may also promote microbial inorganic P assimilation, leading to P deficiency, though the underlying mechanisms remain unclear. BDF2 increased the contents of SOM, NH_4_^+^-N, water-soluble calcium, and magnesium, which may be due to the release of small-molecule organic substances during degradation, supplementing the soil organic carbon pool [32]. Soil N dynamics are also regulated indirectly by biodegradable plastic films through microbial activities [33]. Film fragments may physically protect soil organic matter from rapid decomposition, further increasing SOM accumulation [34]. However, conflicting findings have also shown that biodegradable film residues can hinder the mineralization of soil organic carbon [32,35], and others have reported that biodegradable plastic films even promote the breakdown of more stable SOM, leading to a decrease in SOM [36]. Combined with increased carbon mineralization, this can also affect the nitrogen cycle due to a low C:N ratio [37]. The divergent nutrient responses to different films highlight the importance of considering film composition and regional environmental conditions when selecting mulching materials.

This study found that there was no significant difference in the impact of film mulching on bacterial diversity, consistent with Samphire et al. [36], indicating that bacteria are relatively resilient to short-term soil environmental changes induced by film application. In contrast, Liu’s study [18] found that degradable film significantly increased soil microbial alpha diversity and argued that it provided a carbon source for certain microorganisms. Changes in soil microbial community structure and function are responses of the soil ecosystem to environmental disturbances [38]. Notably, Shi et al. [39] reported that PBAT exposure reduced taxonomic diversity while increasing functional diversity, suggesting that microbial communities may retain or enhance functional potential even with reduced taxonomic richness. RF treatment increased *Proteobacteria* abundance by 7.66% compared to CK, possibly because polyethylene film promotes soil aggregate formation by regulating soil electrochemical and hydrological properties, providing attachment carriers and carbon sources for *Proteobacteria* (copiotrophic bacteria) [40]. BDF2, however, had lower *Proteobacteria* abundance than CK, likely due to its rapid degradation, reducing microplastic accumulation and minimizing selective pressure favoring plastic-degrading bacteria. *Acidobacteriota* (oligotrophic bacteria) showed a decreasing trend in film mulching treatments, which contradicts their typical negative correlation with SOM [41]. This suggests that in our study, *Acidobacteriota* abundance may be more strongly influenced by other factors such as soil humidity and nutrient ratios. Fungi—particularly Ascomycota and Basidiomycota—are efficient decomposers of complex organic matter and plastics [42], and the increased carbon input from BDF2 degradation likely supports a more diverse fungal community. These compositional shifts suggest that different mulching films may drive distinct soil functional pathways. The increase in fungal decomposers under BDF2 indicates a greater potential for the breakdown of complex organic substrates, while the enrichment of copiotrophic *Proteobacteria* under RF suggests faster turnover of labile carbon pools. Therefore, the observed microbial community changes are likely to reflect shifts in dominant soil carbon transformation processes rather than merely taxonomic differences.

Soil water-soluble magnesium was revealed to be the key factor driving changes in microbial community structure. SWMg was significantly negatively correlated with seven bacterial phyla and positively correlated with five phyla. The higher water-soluble magnesium content in the BDF2 treatment may inhibit the growth of *Proteobacteria* and promote the reproduction of *Acidobacteriota*, thereby altering soil biogeochemical cycling [41]. Reductions in copiotrophic bacterial groups may slow the turnover of readily available carbon, whereas increases in oligotrophic or fungal groups may favor the decomposition of more complex substrates. These functional shifts suggest that film-induced changes in soil nutrient availability can cascade into altered carbon and nutrient cycling dynamics. Ascomycota (the dominant fungal phylum) was significantly positively correlated with AK, possibly because AK promotes the activity of fungal enzymes involved in nutrient cycling. These correlations reflect the adaptive differentiation of microbial communities to soil nutrient gradients.

The film degradation rate is a core factor determining its long-term impact on the soil ecosystem. In this study, BDF2 (PBAT + PLA) exhibited good biodegradability, probably because the addition of PLA accelerates the hydrolysis and microbial degradation process of the material [40]. BDF1 (PBAT + starch) had a slower degradation rate, as the low compatibility between PBAT and starch delays the overall degradation process [43]; RF, composed of polyethylene with a stable molecular structure, was difficult to decompose by microorganisms in the natural environment. From the ecological perspective, despite BDF2’s reduced heat preservation performance in the later growth stage due to rapid degradation, it improved soil fertility by supplementing SOM and increasing soil air permeability. However, BDF2’s poor humidity retention may lead to hydrothermal stress in southern China’s subtropical humid region, requiring optimization with drip irrigation measures. Although RF has excellent heat preservation and humidity retention properties, long-term use may lead to microplastic accumulation and accelerated SOM decomposition, potentially depleting the soil carbon pool.

From an ecological process perspective, the effects of different mulching films on microbial communities can be interpreted as responses to film-induced changes in soil resource availability and environmental conditions. Conventional polyethylene film mainly modifies soil temperature and moisture regimes, creating more stable and nutrient-rich microenvironments that favor fast-growing, copiotrophic bacterial groups. In contrast, rapidly degradable films such as BDF2 supply additional organic substrates and alter nutrient stoichiometry, promoting fungal decomposers and reshaping bacterial community composition. These shifts in microbial functional groups indicate corresponding changes in soil processes, including organic matter decomposition pathways, carbon turnover rates, and nutrient cycling dynamics. However, the long-term ecological effects of mulching films remain uncertain, as film thickness and material properties may influence microbial communities [44], and biodegradable films may release metal or metalloid additives during degradation [45,46]. Therefore, long-term monitoring and environmental risk assessment are necessary to ensure the sustainable use of biodegradable mulches.

## 5. Conclusions

This study evaluated different plastic film mulches for broccoli cultivation in southern China’s subtropical humid region. RF (reinforced polyethylene) showed the most stable heat preservation and increased available P and K, suiting short-term high yield. BDF2 (PBAT + PLA) significantly improved soil organic matter, NH_4_^+^-N, and water-soluble Ca/Mg, enhanced fungal diversity, reduced potential pathogen abundance, and degraded fastest, favoring sustainable cultivation. BDF1 (PBAT + starch) had the best humidity retention but no significant nutrient or microbial effects. Water-soluble Mg was the key driver of microbial community changes. BDF2 with supplementary drip irrigation is recommended for green cultivation, with future research focusing on optimizing biodegradable film degradation rates and thickness consistency. Future research should include long-term trials, use films of consistent thickness, and integrate functional assays and material modification techniques to improve the applicability and ecological safety of biodegradable films.

## Figures and Tables

**Table 1:** Characteristics of the tested plastic films.

Name	Type	Main Components	Color	Thickness /mm	Source
RF	Reinforced film	Polyethylene	Black	0.018	Shandong Weifang Changle Tianhe Plastic Industry Co., Ltd., Weifang, Shandong, China.
BDF1	Biodegradable film	PBAT + starch	Black	0.01	Changzhou Bailiji Biodegradable Materials Co., Ltd., Changzhou, Jiangsu, China.
BDF2	Biodegradable film	PBAT + PLA	Black	0.01	Shanghai Heruifeng New Materials Technology Co., Ltd., Shanghai, China.

**Table 2:** Soil properties under different plastic film treatments.

Index	Ctr	RF	BDF1	BDF2
pH	8.11 ± 0.11 ^a^	8.14 ± 0.13 ^a^	8.19 ± 0.11 ^a^	8.04 ± 0.11 ^a^
SOM (g/kg)	17.15 ± 1.77 ^ab^	15.65 ± 2.37 ^ab^	15.86 ± 2.01 ^ab^	19.54 ± 1.83 ^a^
NO_3_^−^-N (mg/kg)	3.18 ± 0.96 ^a^	4.36 ± 0.6 ^a^	3.61 ± 1.12 ^a^	3.89 ± 0.03 ^a^
NH_4_^+^-N (mg/kg)	2.75 ± 0.06 ^b^	3.09 ± 0.28 ^ab^	2.86 ± 0.56 ^b^	3.53 ± 0.14 ^a^
AP (mg/kg)	27.97 ± 2.9 ^b^	33.24 ± 3.05 ^a^	27.56 ± 3.13 ^b^	34.35 ± 2.53 ^a^
AK (mg/kg)	61.17 ± 4.62 ^bc^	99.50 ± 7.0 ^a^	58.50 ± 1.0 ^c^	81.50 ± 3.61 ^b^
AFe (mg/kg)	10.09 ± 2.28 ^a^	11.69 ± 1.63 ^a^	12.07 ± 1.42 ^a^	9.67 ± 1.34 ^a^
AMn (mg/kg)	2.06 ± 0.17 ^a^	1.68 ± 0.47 ^a^	2.19 ± 0.41 ^a^	2.05 ± 0.11 ^a^
ACu (mg/kg)	1.53 ± 0.16 ^a^	1.56 ± 0.07 ^a^	1.49 ±0.15 ^a^	1.49 ± 0.07 ^a^
AZn (mg/kg)	7.42 ± 1.66 ^ab^	6.40 ± 1.42 ^ab^	5.97 ± 0.84 ^b^	8.81 ± 1.77 ^a^
AB (mg/kg)	0.49 ± 0.09 ^a^	0.51 ± 0.10 ^a^	0.48 ± 0.10 ^a^	0.53 ± 0.11 ^a^
AMo (mg/kg)	0.05 ± 0.005 ^ab^	0.06 ± 0.01 ^a^	0.04 ± 0.003 ^b^	0.04 ± 0.11 ^b^
Wa-Ca (mg/kg)	49.33 ± 5.02 ^b^	64.48 ± 1.27 ^a^	65.64 ± 1.97 ^a^	66.11 ± 1.11 ^a^
Wa-Mg (mg/kg)	10.88 ± 1.58 ^b^	15.18 ± 3.75 ^ab^	15.30 ± 3.08 ^ab^	18.30 ± 1.08 ^a^

Different lowercase letters after data in the same column indicate significant differences between treatments (*p* < 0.05).

**Table 3:** Soil microbial alpha diversity indices under different treatments.

Estimators	Ace	Chao	Coverage	Shannon	Simpson	Sobs
Ctr	6777 ± 287.2 ^a^	6518 ± 286.6 ^a^	0.9385 ± 0.0029 ^a^	7.282 ± 0.077 ^a^	0.0019 ± 0.0003 ^a^	4556 ± 132.4 ^a^
RF	6768 ± 339 ^a^	6459 ± 297.6 ^a^	0.9389 ± 0.0034 ^a^	7.3 ± 0.073 ^a^	0.0021 ± 0.0003 ^a^	4622 ± 214.1 ^a^
BDF1	6768 ± 229.9 ^a^	6514 ± 280.4 ^a^	0.9388 ± 0.0022 ^a^	7.322 ± 0.1 ^a^	0.0019 ± 0.0003 ^a^	4649 ± 133.1 ^a^
BDF2	6729 ± 313.6 ^a^	6460 ± 253.8 ^a^	0.9384 ± 0.0023 ^a^	7.037 ± 0.436 ^a^	0.0087 ± 0.0118 ^a^	4515 ± 199.5 ^a^
Ctr	473.6 ± 103.4 ^abc^	474.3 ± 113.2 ^ab^	0.9981 ± 0.0003 ^b^	3.37 ± 0.77 ^a^	0.12 ± 0.073 ^a^	412.7 ± 102.2 ^ab^
RF	374.1 ± 62.92 ^c^	374.7 ± 62.15 ^b^	0.9992 ± 0.0002 ^a^	3.702 ± 0.59 ^a^	0.095 ± 0.064 ^a^	355.7 ± 64.14 ^b^
BDF1	452.6 ± 15.46 ^abc^	455.9 ± 18.12 ^ab^	0.9986 ± 0.0002 ^ab^	3.429 ± 0.761 ^a^	0.141 ± 0.119 ^a^	421.3 ± 17.04 ^ab^
BDF2	520.3 ± 67.28 ^ab^	532.9 ± 87.67 ^a^	0.9985 ± 0.0006 ^ab^	3.781 ± 0.475 ^a^	0.084 ± 0.052 ^a^	483 ± 50.74 ^a^

Different lowercase letters after data in the same column indicate significant differences between treatments (*p* < 0.05).

**Table 4:** Correlation between dominant bacterial phyla and soil parameters.

Phylum	pH	SOM	NO_3_^−^-N	NH_4_^+^-N	AP	AMn	AZn	AB	AMo	Mg
*Proteobacteria*	0.14	−0.59 *	−0.01	−0.62	−0.27	0.03	0.14	−0.11	0.27	−0.91 ***
*Acidobacteriota*	−0.46	0.62 *	0.06	0.27	0.26	0.06	−0.38	−0.20	−0.08	0.72 **
*Actinobacteriota*	0.40	−0.16	0.07	0.06	−0.07	−0.01	0.52	−0.22	−0.14	−0.63 *
*Firmicutes*	−0.25	−0.12	0.58 *	0.39	0.49	0.20	0.57	0.39	−0.32	−0.10
*Gemmatimonadota*	0.66 *	−0.55	−0.24	−0.47	−0.50	−0.24	0.05	−0.34	0.31	−0.76 **
*Bacteroidota*	0.14	0.02	−0.32	−0.26	−0.05	0.57	0.40	0.00	−0.35	−0.62 *
*Methylomirabilota*	−0.07	−0.01	−0.23	−0.37	−0.33	−0.32	−0.40	−0.70 *	0.40	−0.09
*Myxococcota*	0.62 *	−0.29	−0.25	−0.14	−0.28	−0.27	0.14	−0.38	0.12	−0.56
*Cyanobacteria*	−0.41	0.57	0.14	0.39	0.22	0.29	0.24	0.29	−0.63	0.54
*Nitrospirota*	0.31	−0.34	−0.14	−0.57	−0.61	−0.45	−0.17	−0.27	0.66 *	−0.54
*Desulfobacterota*	0.45	−0.18	−0.15	−0.27	−0.47	−0.61 *	−0.43	−0.32	0.63 *	−0.12
*Planctomycetota*	−0.37	0.49	−0.08	0.22	0.13	−0.13	−0.46	−0.29	−0.02	0.75 **
*Latescibacterota*	−0.31	0.28	−0.11	−0.17	0.04	−0.06	−0.76 **	−0.27	0.29	0.48
*Verrucomicrobiota*	−0.40	0.23	0.19	0.04	0.11	−0.27	−0.76 **	−0.11	0.23	0.62 *
NB1-j	0.09	−0.34	−0.22	−0.41	−0.32	−0.54	−0.44	−0.54	0.64 *	−0.22
*Patescibacteria*	0.35	−0.45	−0.06	−0.34	−0.14	0.07	0.06	−0.05	0.10	−0.73 **

* *p* < 0.05, ** *p* < 0.01, *** *p* < 0.001.

**Table 5:** Correlation between dominant fungal phyla and soil parameters.

Phylum	pH	SOM	NO_3_^−^-N	NH_4_^+^-N	AP	AK	AFe	AMn	ACu	AZn	AB	AMo	Ca	Mg
*Ascomycota*	−0.30	0.10	0.55	0.22	0.56	0.72 **	0.59 *	0.17	0.25	−0.31	0.03	0.17	0.43	0.19
*Basidiomycota*	−0.32	0.23	−0.16	0.08	−0.16	−0.39	−0.38	0.19	−0.27	0.63 *	−0.22	−0.63 *	−0.27	−0.15
*Chytridiomycota*	0.06	0.47	−0.18	0.21	0.20	0.07	0.03	0.13	−0.13	−0.54	0.07	−0.06	0.17	0.61 *
*Fungi_phy_* *Incertae_sedis*	−0.19	0.19	−0.16	−0.42	−0.02	−0.20	0.22	0.26	0.34	−0.66 *	0.26	0.14	−0.15	0.19
*Glomeromycota*	−0.05	0.09	0.32	0.19	0.53	0.46	0.59 *	0.29	0.27	−0.13	0.18	−0.08	0.43	0.07
*Zoopagomycota*	−0.33	0.27	0.23	0.50	0.67 *	0.43	0.35	0.41	0.17	0.01	−0.07	−0.40	0.47	0.54
*Blastocladiomycota*	−0.04	0.12	−0.21	−0.25	0.07	−0.02	0.22	0.30	0.15	−0.59 *	0.67 *	0.34	−0.02	0.33
*Kickxellomycota*	−0.75 **	0.07	0.58 *	0.29	0.57	0.53	0.41	−0.05	0.41	−0.10	−0.35	−0.10	0.39	0.30
*Monoblepharomycota*	−0.2 5	0.17	0.13	0.12	0.07	−0.03	−0.05	0.25	−0.12	0.75 **	−0.11	−0.48	0.18	−0.33
*Aphelidiomycota*	−0.67 *	0.59 *	0.31	0.26	0.23	0.10	−0.14	0.16	−0.09	0.05	0.26	−0.27	0.38	0.45
*Basidiobolomycota*	0.19	0.21	−0.44	−0.32	−0.64 *	−0.56	−0.61 *	−0.43	−0.29	−0.31	−0.23	0.18	−0.48	−0.06
*Mucoromycota*	−0.38	0.05	0.16	−0.08	−0.07	0.22	−0.11	−0.51	0.09	−0.65 *	−0.29	0.61 *	−0.01	0.36

* *p* < 0.05, ** *p* < 0.01.

## Data Availability

The original contributions presented in this study are included in the article. Further inquiries can be directed to the corresponding authors.
